# Trichoscopy of Androgenetic Alopecia: A Systematic Review

**DOI:** 10.3390/jcm13071962

**Published:** 2024-03-28

**Authors:** Agata Kuczara, Anna Waśkiel-Burnat, Adriana Rakowska, Małgorzata Olszewska, Lidia Rudnicka

**Affiliations:** Department of Dermatology, Medical University of Warsaw, Koszykowa 82a, 02-008 Warsaw, Poland

**Keywords:** androgenetic alopecia, dermoscopy, hair loss, pattern hair loss, videodermoscopy

## Abstract

**Background**: Androgenetic alopecia, the most common cause of non-scarring hair loss, is a consequence of the gradual miniaturization of the hair follicles. In the majority of male androgenetic alopecia cases, a patient’s history and clinical evaluation may be sufficient to establish the diagnosis, while for women, they should be supplemented with trichoscopy. **Methods**: The PubMed and Scopus databases were used to collate published studies and to analyze the most typical trichoscopic findings in patients diagnosed with androgenetic alopecia. A total of 34 articles were retrieved after exclusion. **Results**: The most common features identified using trichoscopy included hair diameter variability (94.07% of patients), vellus hairs (66.45%) and the peripilar sign (43.27%). Others, such as the honeycomb pattern, yellow and white dots, were less relevant. **Conclusions**: We concluded that hair diameter variability, vellus hairs and the peripilar sign represented valuable indicators for the diagnosis of androgenetic alopecia.

## 1. Introduction

Androgenetic alopecia (AGA), also known as pattern hair loss, is the most common cause of non-scarring alopecia. It affects 50% of women and 80% of men with the onset at adolescence in all ethnicities [1]. It is caused by an interaction between androgens, their metabolites and genetic predisposition. Specifically, a conversion of testosterone to dihydrotestosterone within the dermal papilla causes the progressive miniaturization of the hair follicles, hair thinning and the development of alopecia [2]. The factors involved in the pathogenesis include the microinflammation of the follicular bulge, the abnormal sensitivity of hair follicles to circulating androgens and irregularities in the arrector pili muscles [3]. The reduction in hair density may also be attributed to the extension of the kenogen phase, occurring concurrently with the miniaturization process of the hair follicle in the frontoparietal region [4].

The clinical presentation of AGA is different in men and women. Typically, hair thinning in the frontotemporal areas, the recession of the frontotemporal hairline and hair loss in the vertex area occur in male androgenetic alopecia (MAGA). In female androgenetic alopecia (FAGA), hair thinning occurs over the frontal and parietal areas of the scalp (Ludwig type) [1,5] or only in the central part of the frontal area (Olsen type, alternatively known as the “Christmas tree pattern”) [6].

There are few classification systems for grading purposes. The Hamilton–Norwood classification system for men and a three-point scale—the Ludwig classification system—for women are the most widely used to characterize hair loss patterns [7]. The diagnosis of AGA is usually based on clinical appearance, but in doubtful cases, trichoscopy may help physicians to make a proper diagnosis and avoid other invasive diagnostic methods such as scalp biopsy [8].

The term trichoscopy was first coined by Rudnicka and Olszewska in 2006 [9]. This non-invasive technique based on dermoscopy may improve the accuracy of the diagnosis of hair diseases. During trichoscopy, images of the hair shafts in the frontal, occipital and both temporal areas are typically examined at 20- or even up to 1000-fold magnifications [10]. Trichoscopy is also used to perform measurements and to assess the disease activity, severity and prognosis, as well as to adjust the therapeutic response [11].

This study employs curated data from prior research to pinpoint the most prominent trichoscopic features aiding in the swift identification of patients with AGA and consequently enhancing the efficacy of practitioners.

## 2. Materials and Methods

This review is a summary of trichoscopic findings in patients with AGA based on data collected by searching the PubMed and Scopus databases using the following terms: “androgenetic alopecia”, “hair loss pattern” combined with “trichoscopy” or “dermoscopy” or “dermatoscopy” or “videodermoscopy” or “videodermatoscopy”. The search was performed on 12 December 2023.

**Figure 1:**
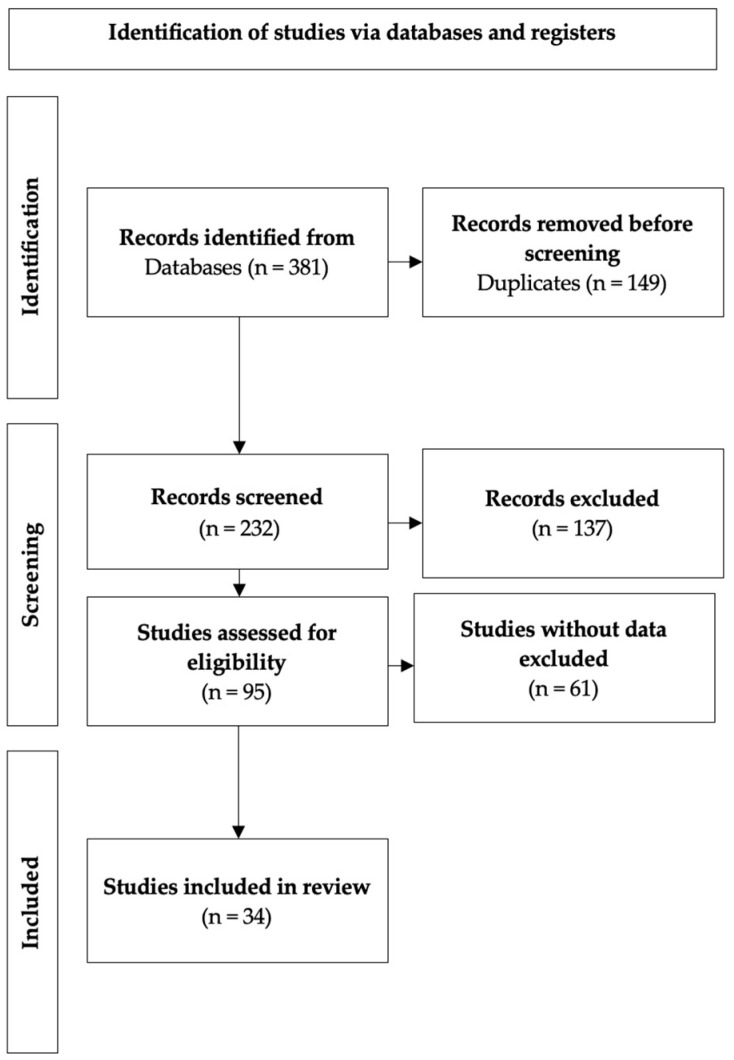
The PRISMA flowchart.

In total, 381 articles were retrieved. Out of those, 149 were identified as duplicates. This resulted in a dataset of 232 articles that were manually analyzed. Subsequently, case reports, animal studies, book chapters and guidelines were excluded, as well as any articles published in a language other than English. Moreover, if the epidemiologic data were incomplete, such as a missing number of patients or an unknown frequency of trichoscopic findings, the research was ignored. Additionally, if a study used both a polarized and a nonpolarized dermoscope, the findings for the former were used. Applying such a procedure yielded a total of 34 articles that were used to compute the necessary data to perform the systematic review (Figure 1).

The collected dataset featured a total number of 2860 patients with AGA and 1840 patients from control groups, either healthy or with other potential hair disorders. Based on this information, the mean values, specificity, sensitivity, the positive predictive value and negative predictive value were calculated. To calculate statistics for a given feature, only studies where it was examined were used. Additionally, for the computation of the predicted values, the prevalence of AGA was considered. The summary of the data is presented in Table 1 and Table 2.

## 3. Results

The review identified 34 articles, which were subjected to meticulous selection and were subsequently incorporated into the quantitative analysis, as delineated in the Preferred Reporting Items for Systematic Reviews and Meta-Analyses (PRISMA) flow diagram in Figure 1. In total, 2860 patients diagnosed with androgenetic alopecia (AGA) were included in the systematic review, with 1295 men and 1565 women constituting this group of subjects. The results of the quantitative analysis, presented in Table 1 and Table 2, are expounded upon below.

**Figure 2:**
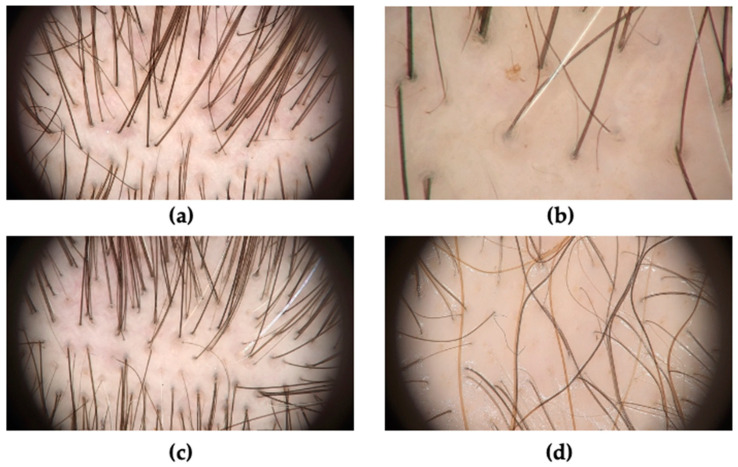
(**a**) Hair shaft heterogeneity with the loss of triple-hair units and yellow dots. (**b**) Vellus hairs. (**c**) Early-stage AGA with peripilar signs. (**d**) Late-stage AGA with the loss of single-hair units and numerous yellow dots.

Hair shaft thickness heterogeneity (Figure 2a) is a typical trichoscopic sign of AGA. In the majority of studies included in the analysis, a hair shaft thickness heterogeneity over 20% in men and over 10% in women was considered as a diagnostic criterion for AGA. However, in some publications, the definition of hair diameter diversity was not determined.

The variation in hair thickness was studied in 27 out of 34 articles. The incidence rate varied between 48.40% and 100% with the mean value of 94.07%. The finding was observed in 99.81% of men and 89.82% of women (Table 1). In women, it was positively correlated with the severity of AGA. Hair shaft thickness heterogeneity was seen in 85.35% of Ludwig 1, in 99.37% of Ludwig 2 and in 100% of Ludwig 3 stage (Table 2). The sensitivity of this finding was the highest among all the analyzed features (94.07%). The positive predictive value was equal to 95.99% (Table 1).

Vellus hairs (Figure 2b) were less commonly studied in patients with AGA compared to the other trichoscopic features. They were described in 13 articles and examined in 775 patients.

The mean frequency of vellus hairs was 66.45%, 61.25% and 78.19% for the total number of AGA patients, men and women, respectively (Table 1). The sensitivity was equal to 66.45%, while the specificity was 65.39%. Moreover, the positive and negative predictive values were calculated to be 56.97% and 58.80%, respectively (Table 1). No study concerning the frequency of vellus hairs depending on the severity of the disease was found.

The peripilar sign (Figure 2c), also known as perifollicular hyperpigmentation, was studied in 24 out of 34 selected articles. Based on our results, the mean frequency of the peripilar sign in patients with AGA was 43.27% with the incidence rate between 9.70% and 100%. The peripilar sign was more common in men (63.67%) than women (42.53%) (Table 1). The analysis showed that the calculated specificity was the highest among all the features (96.06%). The positive predictive value was also considerable at 95.76% (Table 1).

Yellow dots (Figure 2a,d) were analyzed in 22 out of 34 selected studies. The incidence rate of yellow dots in patients with AGA varied between 2.90% and 91.20%, while the mean value was 27.30% (Table 2). The specificity and sensitivity were estimated at 74.45% and 27.30%, respectively. Additionally, the positive predictive value was 62.31% for this feature, while the negative predictive value was 63.80% (Table 2).

The honeycomb pattern was studied in 17 articles. The mean frequency of this feature in AGA was calculated at 32.33% and the incidence rate at 0–85.70%. The calculations revealed a correlation between the severity of the disease and the presence of the honeycomb pattern in women (Table 2). The sensitivity was calculated at 32.33%. The specificity was more significant and equal to 84.18%. The positive and negative predictive values were 75.92% and 62.80%, respectively (Table 1).

The occurrence of white dots in patients with AGA was studied in 12 articles. It was reported in 24.97% of cases (28.02% of men and 21.37% of women) with the frequency ranging from 14.70% to 90.00% (Table 1). Additionally, a correlation between the severity of the disease and the occurrence of white dots could be identified, both among male and female patients (Table 2). As regards white dots, the sensitivity was the lowest among the studied trichoscopic features and equal to 24.97%. The specificity was 81.56%. The positive and negative predictive values were estimated at 66.44% and 69.08%, respectively (Table 1).

## 4. Discussion

**Figure 3:**
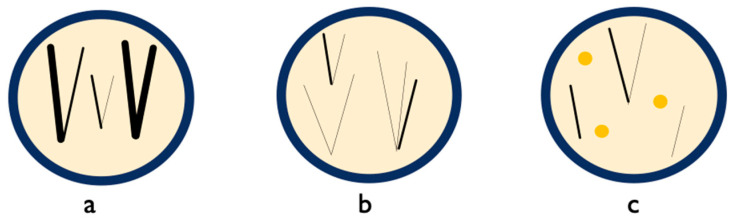
In AGA pathogenesis, the conversion of free testosterone to dihydrotestosterone leads to unsynchronized hair follicle miniaturization, depicted in trichoscopy by varying hair thickness (**a**) and increased number of vellus hairs (**b**), while the androgen inhibition of the anagen phase manifests itself as an increased number of follicular units with one hair and yellow dots (**c**), representing follicular infundibula filled with keratotic material, indicative of hairs in the kenogen phase.

Trichoscopic findings in AGA result from the influence of dihydrotestosterone on the hair follicles (Figure 3).

This systematic review aimed to analyze the most typical trichoscopic findings in patients diagnosed with AGA. The evaluation of the included studies suggested that hair diameter variability, vellus hairs and the peripilar sign were the most relevant in the case of AGA. Our results are consistent with criteria established by Rakowska et al. [11], which allow for diagnosing FAGA based on trichoscopy with 98% specificity. The major criteria include the ratio of over four yellow dots in four images (70-fold magnification) in the frontal area, lower average hair thickness in the frontal area compared to the occiput and more than 10% of thin hairs (below 0.03 mm) in the frontal area. Minor criteria encompass an increased frontal-to-occipital ratio of single-hair pilosebaceous units, vellus hairs and perifollicular discoloration.

Hair shaft thickness heterogeneity, also known as anisotrichosis, is the main trichoscopic feature of AGA. In AGA, the unsynchronized progressive miniaturization of the hair follicles, which corresponds to vellus transformation, is observed in the affected scalp region (Figure 3) [45]. A hair shaft thickness heterogeneity of >10% for women and >20% for men is considered as a diagnostic criterion for AGA [15,16]. In the present analysis, hair shaft thickness heterogeneity was observed in all stages of MAGA, while in women, the frequency of the variation in hair diameter correlated with the severity of the disease.

In the literature, no significant correlation was found between the frequency of the hair shaft thickness heterogeneity and skin phototypes [17]. This study showed that the variation in hair thickness, which was observed in the early stages of AGA, was a very common indicator of this disease and might be an important criterion for the diagnosis by a physician. Based on a high positive predictive value and specificity, the patients who present such a feature should be considered as suffering from AGA.

By definition, the development of vellus hairs (defined as 0.03 mm or less thick and 2–3 mm long) results from the abnormal sensitivity of hair follicles to androgens and irregularities in the arrector pili muscles (Figure 3) [3]. This feature is currently regarded as a marker of AGA. Vellus hairs are more visible during dry dermoscopy, without using an immersion fluid. The presence of vellus hairs in the frontal hair line is useful in the differentiation between FAGA and frontal fibrosing alopecia [46]. Rakowska et al. [11] determined two vellus hairs in the frontal area to be normal in healthy individuals at 20-fold magnification. According to Rakowska et al. [11], over 10% of thin hairs (<0.03 mm) in the frontal area is a characteristic feature of FAGA. In our analysis, vellus hairs were present in 66.45% of the subjects with AGA and were more common in women than in men. Moreover, this trichoscopic feature has a relatively high sensitivity which is equivalent to the low chance of a false negative result. Nevertheless, the results for vellus hairs were calculated using data from fewer articles and patients compared to other studies, which has an impact on their interpretability.

A conspicuous brown halo, also known as the peripilar sign, indicates perifollicular inflammation [13]. It was suggested that this trichoscopic feature was mainly observed in the early stages of AGA. However, our analysis did not confirm such a hypothesis. Studies showed that this feature was more commonly observed in Caucasian AGA patients compared to Asian ones. This may be due to the skin color of Asian patients which conceals them [18,47]. In this study, the peripilar sign was found to be the trichoscopic feature with the highest specificity and a significant positive predictive value. Therefore, its presence strongly suggests considering AGA as a potential diagnosis. However, it should be assessed by taking account of the patient’s clinical condition and other trichoscopic features. Additionally, the absence of this symptom should not stop physicians from further diagnostics for AGA based on its average occurrence among patients.

Yellow dots were first described in 2006 by Ross et al. [40] as a feature typically observed in alopecia areata. They are the follicular infundibula filled with keratotic material or/and sebum. In AGA, their occurrence is associated with the kenogen phase (Figure 3) [48]. Yellow dots in AGA are solitary and predominate in the frontal area of the scalp [49]. Their size and distribution are more irregular, and they are distributed between normal-looking hairs. The number of the features is generally lower than in alopecia areata [49]. Based on Rakowska et al. [11], the presence of at least four yellow dots in the frontal area of the scalp on trichoscopic examination is one of the major criteria to identify FAGA. Consequently, it helps to limit the use of more invasive testing approaches. Based on our results, AGA should be considered as a diagnosis in individuals with yellow dots on trichoscopic examination. It is supported by a relatively high specificity and a positive predictive value. Conversely, as the sensitivity is relatively low, the absence of such a feature should not exclude AGA. It is worth mentioning that our study has limitations. The control group includes patients with other hair disorders as well, which impedes the interpretation of the results as it is commonly known that this feature may occur in a number of other conditions.

The honeycomb pattern is a hyperchromic ring on the skin surface resulting from sun exposure in a thinning or completely hairless area. Therefore, the feature is more pronounced in individuals with darker skin phototypes, typically in men with advanced AGA [8]. Based on a study by Kibar et al. [19], the honeycomb pigment pattern was found to be more common in severe MAGA. Moreover, Hu et al. [13] and Zhang et al. [44] suggested a positive correlation with AGA stages, in contrast to a study by Ummiti et al. [15], who declined such an observation. The honeycomb pattern is not very common in patients with AGA (32.33%). However, its high predictive value and specificity suggest that the occurrence of this feature may prove very useful in making a diagnosis. However, as with the peripilar sign, it should always be interpreted along with the patient’s clinical status.

White dots may be observed as fibrotic or pinpoint white dots. The former may be seen as a pearly feature, especially in more advanced cases of AGA. They are better visualized in dark-skinned patients. Tawfik et al. [17] also suggested that fibrotic white dots together with the white peripilar sign could be used as a marker to diagnose mild fibrosis in patients with long-lasting AGA. Pinpoint white dots correspond to eccrine sweat duct openings in the scalp and represent hypertrophic sebaceous glands [8]. Furthermore, both features are more pronounced on the hyperpigmented scalp, which may explain why they are more common in severe cases. Abraham et al. [50] reported that white dots were also found in healthy patients with Fitzpatrick IV, V and VI skin phototypes. This feature is rarely observed in patients with AGA. However, because of the high specificity and a positive predictive value, it may help diagnose AGA. Additionally, the presence of white dots was correlated with the severity of the disease, which is consistent with the previous findings by Tawfik et al. [17].

Other parameters evaluated in the selected studies included empty follicle, single- or double-hair units and black dots, with 16 studies focusing on the former and 5 studies on the latter. While the literature on the empty follicle, single- or double-hair units is substantial, their inclusion in the review was precluded due to inconsistencies and inadequacies in monitoring methodologies. Additionally, black dots were excluded from the statistical analysis, as the available data were insufficient to derive conclusive findings.

In healthy individuals, hairs grow as groups of several hair roots in one follicular orifice [51]. Rakowska et al. [51] established normal values of the percentage of pilosebaceous units with a single hair at below 35% in the frontal area, 30% in the occiput and 40% in the temporal areas. On the contrary, a ratio of single-hair unit percentage in the frontal area to the occiput > 2:1 is necessary to diagnose FAGA [11]. It was suggested that the loss of triple-hair units could be a very important indicator of early-stage AGA. Additionally, recent studies have shown that the presence of two hairs of different thicknesses in one hair unit is a characteristic feature of AGA [46]. Due to the insufficient data, it was not possible to include this trichoscopic feature in the statistical calculations.

Black dots, which are also described as “cadaveric hairs”, represent fractured dystrophic, pigmented hairs at the scalp level. They are typical of alopecia areata, dissecting cellulitis, trichotillomania and tinea capitis [52]. Some authors reported such a feature in patients with AGA [8,14,38]. However, black dots were only described in four articles, so we decided not to take them into account, as statistical measures could not be effective enough to draw any conclusions.

While the trichoscopic features of androgenetic alopecia are distinguishable, it is important to recognize that there are other diseases that mimic AGA, including lichen planopilaris, telogen effluvium, frontal fibrosing alopecia, fibrosing alopecia in a pattern distribution and traction alopecia. Table 3 [46,53,54,55,56] provides examples of these conditions along with their trichoscopic features, aiding in their differentiation from AGA.

There are several limitations of our study. Control groups were missing in several publications. Some authors failed to provide the number of patients, some epidemiological data or details concerning the method of trichoscopy, i.e., the lack of information about the area where the examination was performed or what magnification was used. Moreover, different types of control groups were included, which impacts the reliability of the results. Finally, as regards some features, the literature data are still quite limited, so the statistics were calculated in a low number of patients from several studies which may contribute to bias in the results.

To the best of our understanding, this systematic review represents the initial comprehensive analysis encompassing all pertinent studies since 2006 that determines sensitivity and specificity values for various trichoscopic features in AGA patients. From this analysis, fundamental features facilitating the expedited diagnosis of individuals with AGA were identified.

## 5. Conclusions

Although the evidence is limited, hair diameter variability seems to be a simple and accurate parameter that could be easily assessed during trichoscopy, even with a handheld dermoscope. It represents a valuable feature for the diagnosis of AGA. An increased proportion of vellus hairs might be helpful to make a proper diagnosis, but physicians should always be cautious, because such a feature may occur in healthy individuals or indicate severe alopecia areata. Based on the high specificity of the peripilar sign, this trichoscopic finding is indicative of AGA diagnosis. All the above-mentioned trichoscopic features are more prominent in the frontal area compared to the occiput.

## Figures and Tables

**Table 1:** Summary of statistics for different trichoscopic features.

Trichoscopic Feature	Mean Value for Patients with AGA	Mean Value for Patients with MAGA	Mean Value for Patients with FAGA	Sensitivity	Specificity	Positive Predictive Value	Negative Predictive Value
Variation in hair diameter [12,13,14,15,16,17,18,19,20,21,22,23,24,25,26,27,28,29,30,31,32,33,34,35,36,37,38]	94.07% (48.40–100%)	99.81%	89.82%	94.07%	91.41%	95.99%	30.76%
Honeycomb pattern [13,14,15,17,19,20,21,25,26,29,31,34,38,39,40,41,42]	32.33% (0–85.70%)	36.25%	29.64%	32.33%	84.18%	75.92%	62.80%
Peripilar sign [13,14,15,16,17,18,19,20,21,24,25,26,27,28,29,31,34,35,36,37,38,39,42,43]	43.27% (9.70–100%)	63.67%	42.53%	43.27%	96.06%	95.76%	51.92%
Yellow dots [13,14,15,16,17,18,19,20,21,25,26,27,28,29,30,31,34,35,36,37,38,39,40,41,42,43]	27.30% (2.90–91.20%)	28.09%	26.11%	27.30%	74.45%	62.31%	63.80%
White dots [13,14,17,19,20,21,26,29,34,38,42,44]	24.97% (14.70–90.00%)	28.02%	21.31%	24.97%	81.56%	66.44%	69.08%
Vellus Hairs [14,16,20,25,26,30,31,33,34,35,36,37,42]	66.45% (30–100%)	61.25%	78.19%	66.45%	65.39%	56.97%	58.80%

**Table 2:** A summary of mean values for AGA patients with various levels of severity of the disease based on the Hamilton–Norwood grades (H-N) and Ludwig stages (L).

Trichoscopic Feature	H-N1	H-N2	H-N3	H-N4	H-N5	H-N6	H-N7	L1	L2	L3
Variation in hair diameter [13,15,17,19,23,32]	100%	100%	100%	100%	100%	100%	100%	85.35%	99.37%	100%
Honeycomb pattern [13,15,17,19]	50%	55.17%	16.26%	46.56%	65%	78.05%	96.77%	14%	38.82%	52.78%
Peripilar sign [13,15,19,44]	53.57%	53.45%	34.48%	37.02%	43.75%	37.80%	36.11%	26.72%	43.99%	36.70%
Yellow dots [13,15,17,19]	92.86%	55.17%	18.47%	30.53%	31.67%	21.95%	25.81%	17.60%	30.92%	44.44%
White dots [13,17,44]	N/A	8.33%	15.06%	30.65%	44.83%	60%	75.86%	11.06%	30.68%	50%

**Table 3:** Trichoscopic features that are characteristic of diseases clinically mimicking androgenetic alopecia.

Disease	Trichoscopic Features
Androgenetic alopecia	Hair diameter variability in the frontal area
	Peripilar sign
	Vellus hairs
Fibrosing alopecia in a pattern distribution	Perifollicular erythema
	Loss of follicular openings
	Peripilar casts
Frontal fibrosing alopecia	Loss of follicular openings
	Peripilar casts
	Perifollicular erythema
	Presence of lonely hairs
	Absence of vellus hairs
Traction alopecia	Empty hair follicles
	Perifollicular erythema
	Atypical red vessels
	Peripilar casts
	Black dots
	Broken hairs
	Comma and coiled hairs
Telogen effluvium	Tip-pointed regrowing hairs
	Location of trichoscopic features such as hair shaft thickness and number of hairs in follicular units on whole scalp
Lichen planopilaris	Perifollicular erythema
	Peripilar casts
	Loss of follicular openings
	Violaceus areas
	Dystrophic hairs
	Absence of vellus hairs
	Fibrotic white dots

## Data Availability

The data presented in this study are available on request from the corresponding author.

## Abbreviations

AGA	androgenetic alopecia
FAGA	female androgenetic alopecia
MAGA	male androgenetic alopecia
